# Examining the Heterogeneous Genome Content of Multipartite Viruses BMV and CCMV by Native Mass Spectrometry

**DOI:** 10.1007/s13361-016-1348-6

**Published:** 2016-02-29

**Authors:** Michiel van de Waterbeemd, Joost Snijder, Irina B. Tsvetkova, Bogdan G. Dragnea, Jeroen J. Cornelissen, Albert J. R. Heck

**Affiliations:** Biomolecular Mass Spectrometry and Proteomics, Bijvoet Center for Biomolecular Research and Utrecht Institute for Pharmaceutical Sciences, Utrecht University, 3584 CH Utrecht, The Netherlands; Netherlands Proteomics Center, Padualaan 8, Utrecht, 3584 CH The Netherlands; Department of Chemistry, Indiana University, Bloomington, IN 47405 USA; Laboratory for Biomolecular Nanotechnology, MESA+ Institute for Nanotechnology, University of Twente, P.O. Box 217, 7500 AE Enschede, The Netherlands

**Keywords:** Native mass spectrometry, Virus, Genome, Brome Mosaic Virus (BMV), Cowpea Chlorotic Mottle Virus (CCMV), Q-TOF, Orbitrap

## Abstract

Since the concept was first introduced by Brian Chait and co-workers in 1991, mass spectrometry of proteins and protein complexes under non-denaturing conditions (native MS) has strongly developed, through parallel advances in instrumentation, sample preparation, and data analysis tools. However, the success rate of native MS analysis, particularly in heterogeneous mega-Dalton (MDa) protein complexes, still strongly depends on careful instrument modification. Here, we further explore these boundaries in native mass spectrometry, analyzing two related endogenous multipartite viruses: the Brome Mosaic Virus (BMV) and the Cowpea Chlorotic Mottle Virus (CCMV). Both CCMV and BMV are approximately 4.6 megadalton (MDa) in mass, of which approximately 1 MDA originates from the genomic content of the virion. Both viruses are produced as mixtures of three particles carrying different segments of the genome, varying by approximately 0.1 MDA in mass (~2%). This mixture of particles poses a challenging analytical problem for high-resolution native MS analysis, given the large mass scales involved. We attempt to unravel the particle heterogeneity using both Q-TOF and Orbitrap mass spectrometers extensively modified for analysis of very large assemblies. We show that manipulation of the charging behavior can provide assistance in assigning the correct charge states. Despite their challenging size and heterogeneity, we obtained native mass spectra with resolved series of charge states for both BMV and CCMV, demonstrating that native MS of endogenous multipartite virions is feasible.

**Electronic supplementary material:**

The online version of this article (doi:10.1007/s13361-016-1348-6) contains supplementary material, which is available to authorized users.

**Graphical Abstract:**
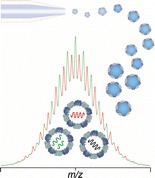
ᅟ

## Introduction

Mass spectrometry of protein complexes under non-denaturing, near-native conditions (native MS) has advanced strongly since the concept was first reported by Brian Chait and co-workers in 1991 [1]. These advances were made possible by important technical developments, mainly through extension of the achievable upper mass (and *m/z*) ranges, the optimization of nanospray ionization sources, and improvements in the mass resolving power. Equally important have been improvements in sample purification and handling protocols, data analysis and interpretation algorithms, and software solutions [2–5]. As such, native mass spectrometry now often becomes integrated in workflows in biology and chemistry (sometimes in combination with other mass spectrometric and structural biology methods) to obtain structural information on proteins and protein complexes [6–14]. Our group as well as others expanded the repertoire of samples measurable from smaller soluble proteins in the kilodalton (kDa) range, through more heterogeneous protein assemblies obtained by affinity purification, to membrane embedded protein complexes and virus capsids in the megadalton (MDa) range [15–19]. Although the limits of what is measurable have been stretched and extended greatly, a number of technical and experimental challenges remain. Notably, the success of analyzing heterogeneous assemblies depends greatly on the size of the system and level of heterogeneity. Endogenous viruses pose both these challenges, as they are not only extremely large (diameter >25 nm, mass >1 MDa) but also commonly exhibit natural heterogeneity because of the genomic or proteinaceous cargo they encapsulate. Although for many viruses the composition of the capsid is known in detail from high-resolution structural analysis with X-ray crystallography or cryo-electron microscopy, the composition of encapsulated nucleic acid or proteins is less well characterized. The encapsulated cargo of a virus particle often does not adhere to the same high order of symmetry as the icosahedral capsid does and variations between individual particles will average out in the analysis. To obtain a more comprehensive understanding of the composition of a virus particle, including details about the encapsulated cargo, orthogonal approaches to high-resolution structural analysis are thus required. Here, we use native mass spectrometry to analyze two closely related endogenous heterogeneous protein–nucleic acid assemblies; the multipartite plant viruses Cowpea Chlorotic Mottle Virus (CCMV) and Brome Mosaic Virus (BMV).

**Figure 1:**
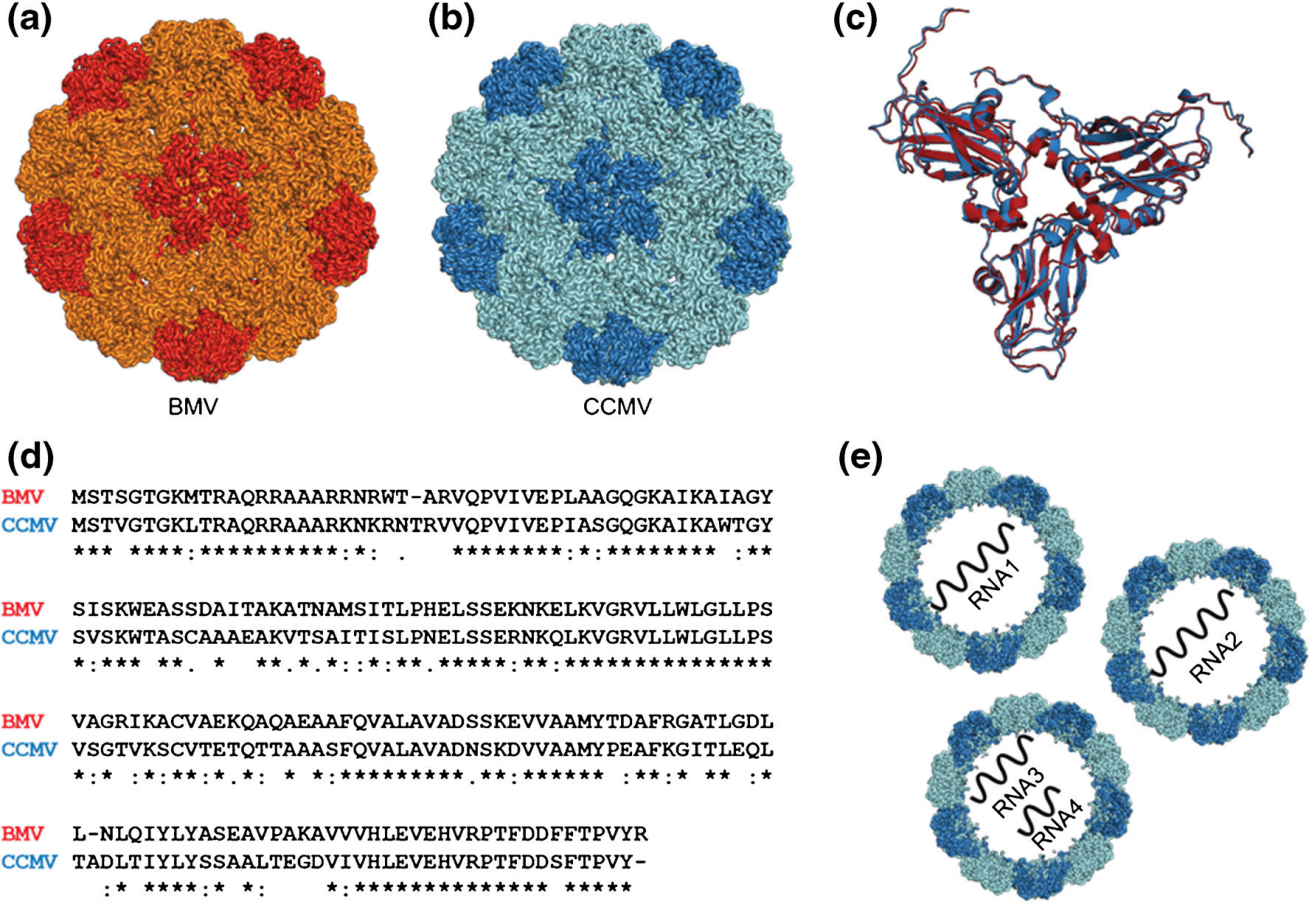
Structural and genomic composition of Brome Mosaic Virus (BMV) and Cowpea Chlorotic Mottle Virus (CCMV). (**a**) Structure of the protein capsid of BMV (based on PDB entry 1JS9) shows high similarity with (**b**) the structure of CCMV (PDB entry 1CWP). (**c**) This similarity becomes even more apparent when the asymmetric trimeric units (BMV in red, CCMV in blue) are overlaid. The characteristic “jellyroll” folds can be seen here. (**d**) Alignment of BMV and CCMV capsid protein sequences shows high homology (70% sequence identity). (**e**) Schematic representation of the genomic architecture of BMV and CCMV. End results of infection are a mixture of particles carrying different segments of the single stranded RNA. RNA1 and RNA2 are packaged separately, whereas RNA3 and RNA4 are co-packaged. All protein structures were rendered using PyMol software

CCMV and BMV share a number of important biochemical and biophysical features (Figure 1). Both belong to the bromoviridae family and contain positive sense single stranded RNA (ssRNA) genomes that are packaged into a non-enveloped icosahedral T = 3 capsid consisting of 180 copies of the capsid protein (Cp_CCMV_ or Cp_BMV_) [20–22]. A main difference between BMV and CCMV is the virus host organism, Cowpea plants for CCMV and grasses for BMV [20]. Their capsid proteins are highly homologous, sharing 70% sequence identity and the masses of the Cp monomers differ less than 50 Da. Both capsid proteins adopt a common “jellyroll” fold and are structurally highly homologous. CCMV and BMV are multipartite viruses, which mean that the end result of infection is a mixture of particles that consist of the same protein capsid encapsulating different segments of the genome (Figure 1e). In both BMV and CCMV, three distinct particles are formed that are all required for infectivity [23, 24]. The viruses package RNA1 and RNA2 separately but co-package RNA3 and a sub-genomic RNA4 [25]. No other proteins are packaged in the virions and infection readily occurs through mechanical inoculation [20, 26]. A segmented genome is commonly believed to provide the opportunity for genetic reassortment, thus increasing diversity [27]. Additionally, multipartite genomes allow viruses to encode more information while keeping the size of the packaging capsid small. Recently, however, Vaughan et al. proposed that partitioning also helps leveraging the differences in the RNA/protein interaction to regulate processes leading to replication [28]. Therefore, the ability of distinguishing between different genomic cargoes encapsulated is an essential first step in pursuing studies aimed at following the fate of each of the particles during the virus life cycle or its laboratory equivalent.

In this paper, we test whether and how we are able to dissect the mass heterogeneity in BMV and CCMV by high resolution native mass analysis making use of both quadrupole time-of-flight (Q-TOF) and Orbitrap mass analyzers and organic additives to manipulate the charging of these assemblies during the electrospray process.

## Experimental

CCMV and BMV were purified after infection in Cowpea and *Nicotiana benthamiana* plants, respectively, as previously described in detail [29–31].

Sample buffers were exchanged to mass spectrometry buffer using centrifuge filter units (Millipore, Darmstadt, Germany). Typically, either 50 mM ammonium acetate at pH 5.0 (CCMV) or 100 mM ammonium acetate pH 7.3 (BMV) was used for mass analysis. For spectra acquired under charge reducing conditions, buffers were the same but with the addition of 25 mM of triethylammonium acetate (TEAA). Small aliquots of the sample (at approximately 2 μM based on the monomeric capsid protein) were infused into the mass spectrometer using nano-electrospray sources consisting of gold-coated borosilicate capillaries that were produced in-house. Native MS experiments were performed on quadrupole time-of-flight (Q-TOF, MS vision; Waters, Manchester, UK) or Orbitrap EMR mass spectrometers (Thermo Scientific, Bremen, Germany) modified for the analysis of high molecular weight proteins [2, 32, 33]. In both instruments, Xenon was used in the collision cell. Typical Q-TOF settings were: 1300–1500 V capillary voltage, 100–200 V cone voltage, 250 V collision energy, 10 mbar source pressure and 2 × 10^–2^ mbar collision gas pressure. Tandem mass spectrometry experiments were acquired with up to 50 V extraction cone voltage and 400 V collision energy. Typical Orbitrap EMR settings were: 1300 V capillary voltage, 200 V extended trapping, 1 × 10^–9^ bar ultra-high vacuum pressure, 250–500 ms injection time, and FT resolution 4000 at 200 *m/z*. Spectra were analyzed using Masslynx 4.1 (Waters, for Q-TOF spectra) or Xcalibur 2.2 Qualbrowser (Thermo Scientific, for Orbitrap EMR data). Instruments were calibrated using CsI clusters.

Simulation of mass spectra was performed using the SOMMS package [34], which generates charge state envelopes for a user-defined envelope width, peak width, mass, and charge state range. In general, the position and width of the charge states envelope were set to match experimental data as much as possible. The peak width was estimated from the experimental data. The relative abundance of the different components was set equally unless the simulated spectra were used to estimate the relative abundances from an experimental spectrum.

## Results and Discussion

The masses of the BMV and CCMV capsid proteins were determined from spectra of denatured monomeric Cp_CCMV_ and Cp_BMV_ to be 20,251 and 20,295 Da, respectively (data not shown). These measured masses hint at the removal of the initiator methionine and N-terminal acetylation for both Cp’s (theoretical masses of 20,254.33 and 20,295.47 Da for Cp_CCMV_ and Cp_BMV_ respectively). No other forms of the capsid proteins could be detected. Based on these data and the expected genome content, we generated a table with the theoretical masses for the three distinct intact BMV and CCMV particles (Table 1).

**Table 1:** Expected Masses for CCMV and BMV Virions. Protein Mass is Based on Assumed Sequences and Processing after Comparison with Masses Obtained by Mass Spectrometry Under Denaturing Conditions. RNA Lengths and Sequences are Taken from the Viper Database. Masses are in Dalton and RNA Lengths in Number of Nucleotides

CCMV					
Particle	Protein copies	Protein mass	RNA length	RNA mass	Total mass
RNA1	180	3645401	3171	1020949	4666351
RNA2	180	3645401	2774	892246.9	4537648
RNA3/4	180	3645401	2173/824	964450.8	4609852
BMV					
Particle	Protein copies	Protein mass	RNA length	RNA mass	Total mass
RNA1	180	3653190	3234	1041798.7	4694989
RNA2	180	3653190	2865	921040.4	4574230
RNA3/4	180	3653190	2111/876	961774.4	4614964

**Figure 2:**
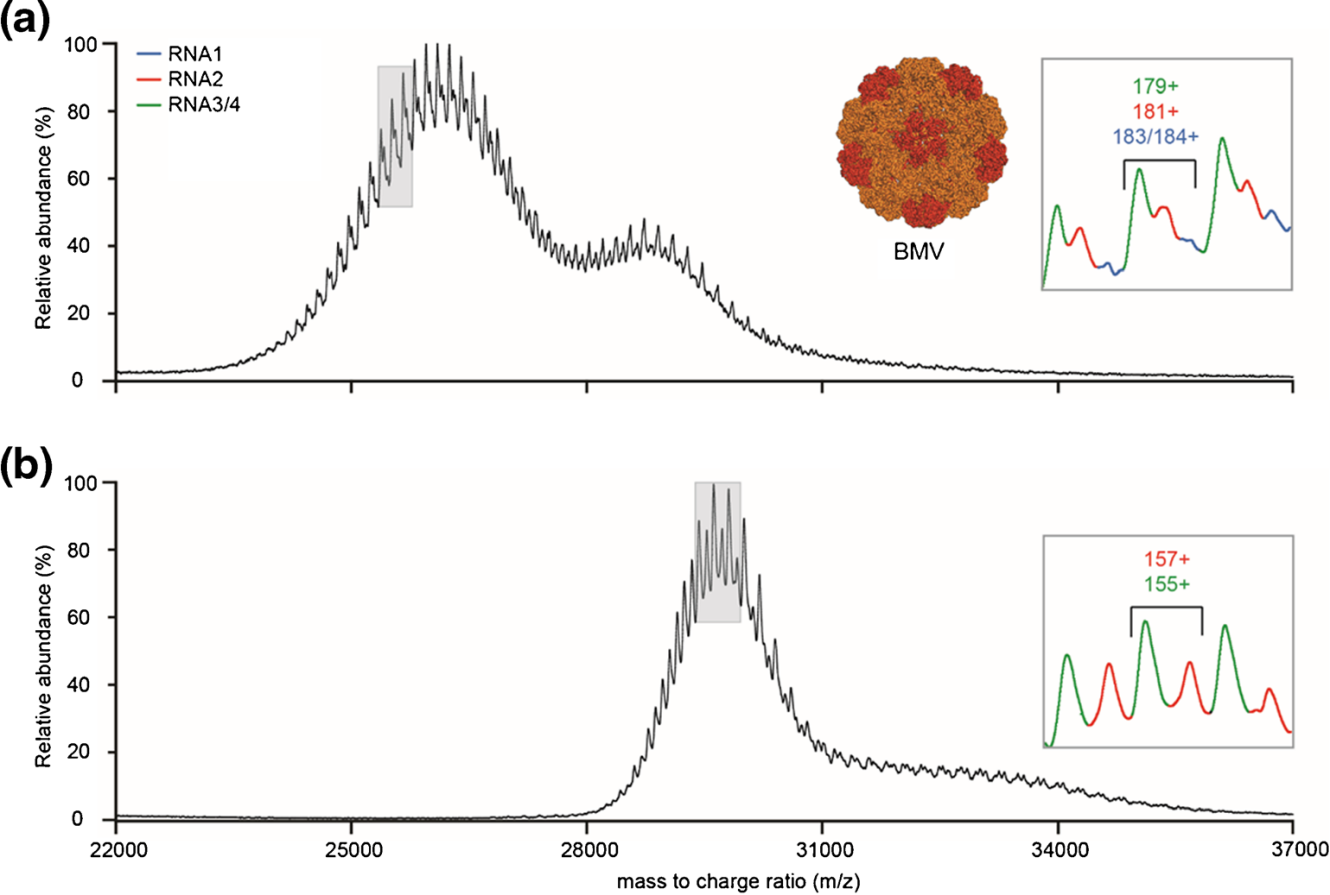
Native mass spectra of BMV endogenous virions acquired on a quadrupole time of flight instrument. (**a**) BMV sprayed from aqueous ammonium acetate and (**b**) sprayed from a mixture of ammonium acetate and triethylammonium acetate. In this particular case, charge reduction through triethylammonium acetate addition lowers the number of particles that are resolved although the spacing between the charge states is increased. Insets: magnified regions of the boxed areas of the spectra, with peaks corresponding to three charge states of the three distinct BMV particles colored. The charge state assignments for the middle charge states are indicated at the top

We initially recorded native mass spectra of BMV on a modified Q-TOF instrument (Figure 2a). The exact *m/z* position of the BMV signal varied strongly between experiments (23,000–28,500 *m/z*) and seemed to be largely dependent on the angle of the electrospray needle relative to the mass spectrometer entrance. This variation is more commonly observed when using Z-spray sources but varies from analyte to analyte. In the majority of the spectra, the most abundant charge state envelopes were present around 26,000 *m/z*. However, in all cases, less abundant charge distributions were detected at higher *m/z*, with approximately 10% lower charge. This signal persisted at low collisional activation, indicating that they are not (merely) a result of initial gas-phase dissociation. As these low abundant distributions are often convoluted by more abundant distributions and dissociation products, we focused our analysis mainly on the higher charged distribution appearing at lower *m/z*. Initial analysis of the spectrum in Figure 2a indicated the presence of three distinct species. Charge state assignment was confident for two of the species, resulting in masses of 4575.2 ± 0.5 and 4618.9 ± 0.7 kDa, which matched well with the expected masses for RNA2 and RNA3 + 4 particles, respectively. The charge state assignment of the third species was more ambiguous, and was determined as either 4710.3 ± 1.3 or 4684.9 ± 1.0 kDa, which corresponds to a charge difference of 1z. Nonetheless, this is in good proximity of the expected mass for an RNA1 filled BMV particle. Based on the measured masses, we simulated spectra for the different species using the software package SOMMS (Supplementary Figures S1 and S2) [34]. This allowed approximation of the relative levels of the three components as 1.5:1:1 (RNA3 + 4:RNA2:RNA1, respectively). This is close to the values obtained from gel electrophoresis densitometry (1.5:1:0.7, Supplementary Figure S4).

**Figure 3:**
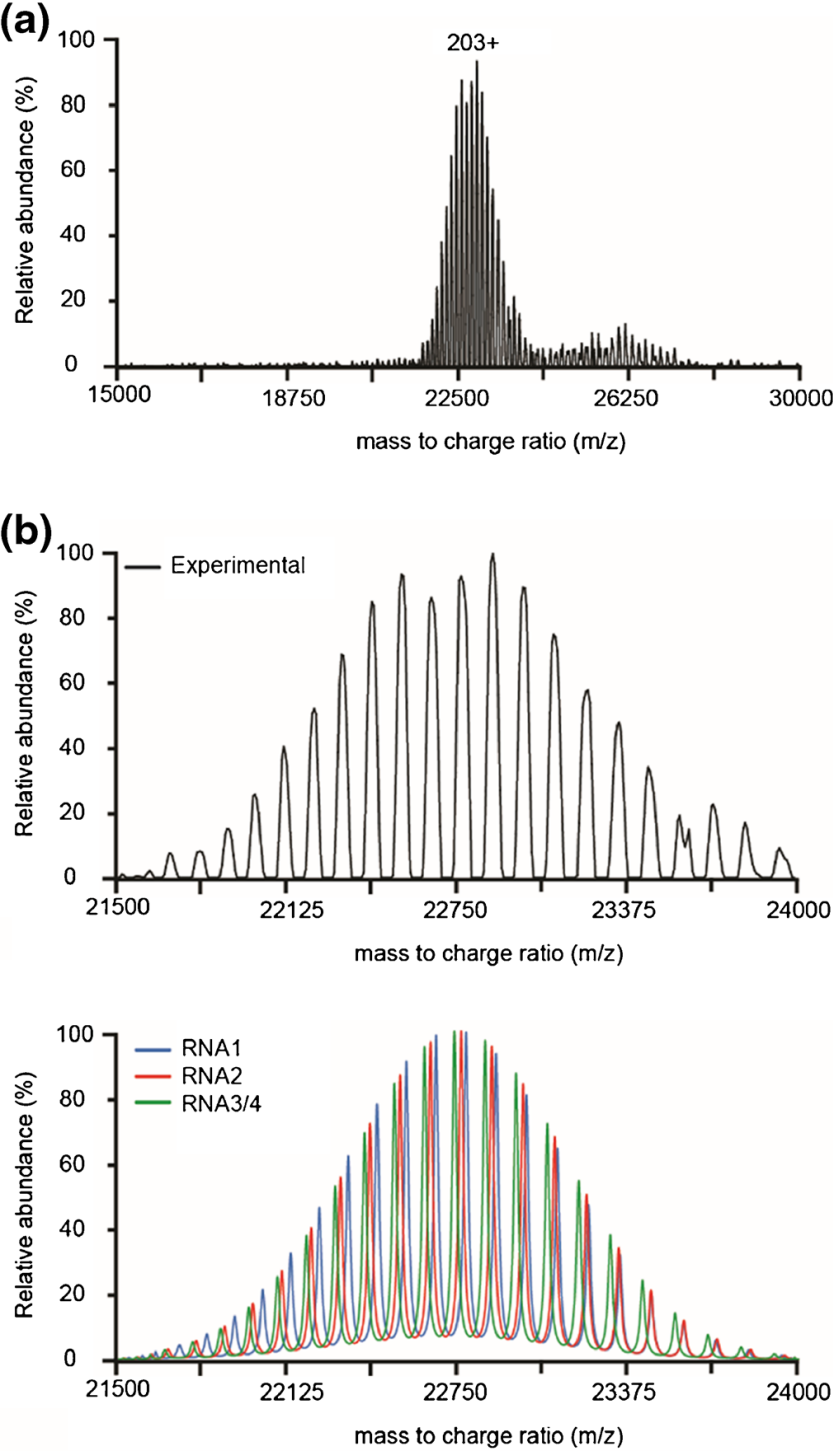
(**a**) Native mass spectra of BMV on the Orbitrap EMR with extended mass range. (**b**) Top: Enlargement of the region around 22,750 *m/z* a shows single series of well-resolved charge states. However, only a single mass could be assigned from this envelope. Bottom: Simulation of the theoretical masses of BMV in the same region indicates close positioning of the peaks belonging to the different BMV particles

We additionally acquired spectra of the BMV virions on the Orbitrap EMR mass spectrometer (Figure 3) [2, 33]. We often observe that large complexes retain more charges when sprayed on the Orbitrap instrument, compared with the Q-TOF instrument. Although we cannot fully explain this, we suspect it to be due to a different design of the source and the different desolvation characteristics in both instruments. The resulting charge state envelope on the Orbitrap centers around 23,000 *m/z* and is clearly base-line resolved, but appeared to contain only a single component related to a particle with a mass of 4621 kDa, which we assigned as the RNA3 + 4 packed BMV virion. The RNA2- and RNA1-containing particles are most likely not resolved at the current instrument resolution because of a combination of factors. First, the retention of more charges causes charge states to be more closely positioned to each other, leaving less free space for multiple components. Second, in the current *m/z* region, the charge states of the three particles are predicted to cluster more closely together in groups of three (see also the simulations in Figure 3b). Finally, the more abundant RNA3 + 4 peaks are predicted to be positioned on the left side of these clusters so any tailing of these peaks can overlap with the RNA1 and RNA2 charge states.

Both the mass measurement and the quantification of the different BMV particles, but in particular the RNA1 virion, are hampered by the significant overlap in the charge states of the different particles. Therefore, we attempted to move the BMV ion signals to higher *m/z* by reducing the charge they attain in the ESI process. A method to reduce the charge states of proteins in the gas phase is through addition of proton-scavenging compounds that have high gas-phase basicity to the spray buffer. Several of these compounds have been reported in literature but have to be used with caution especially when measuring high molecular weight proteins [35, 36]. They are generally not as volatile as the commonly used ammonium acetate and can cause extensive adduct formation, resulting in peak broadening and loss of mass resolving power. To circumvent this, we chose to add 25 mM of triethylammonium acetate (TEAA) prior to injection in the mass spectrometer, as this compound is volatile and has a similar pK_A_ as ammonium acetate. When applied on the Q-TOF instrument, this shifted the center of the charge state distribution to approximately 29,500 *m/z* through a loss of around 12% of the initial charge in ammonium acetate (Figure 2b). In the resulting spectrum, we were able to detect two distinct species and assigned their masses as 4576.9 ± 0.3 and 4620.3 ± 0.3 kDa, in good agreement with the RNA2 and RNA3 + 4 masses. Simulation of the BMV masses indicated that in this *m/z* region, it is most likely not possible to resolve RNA1 from the other two particles, especially since it overlaps mainly with the RNA2 signal (Supplementary Figure S3).

**Figure 4:**
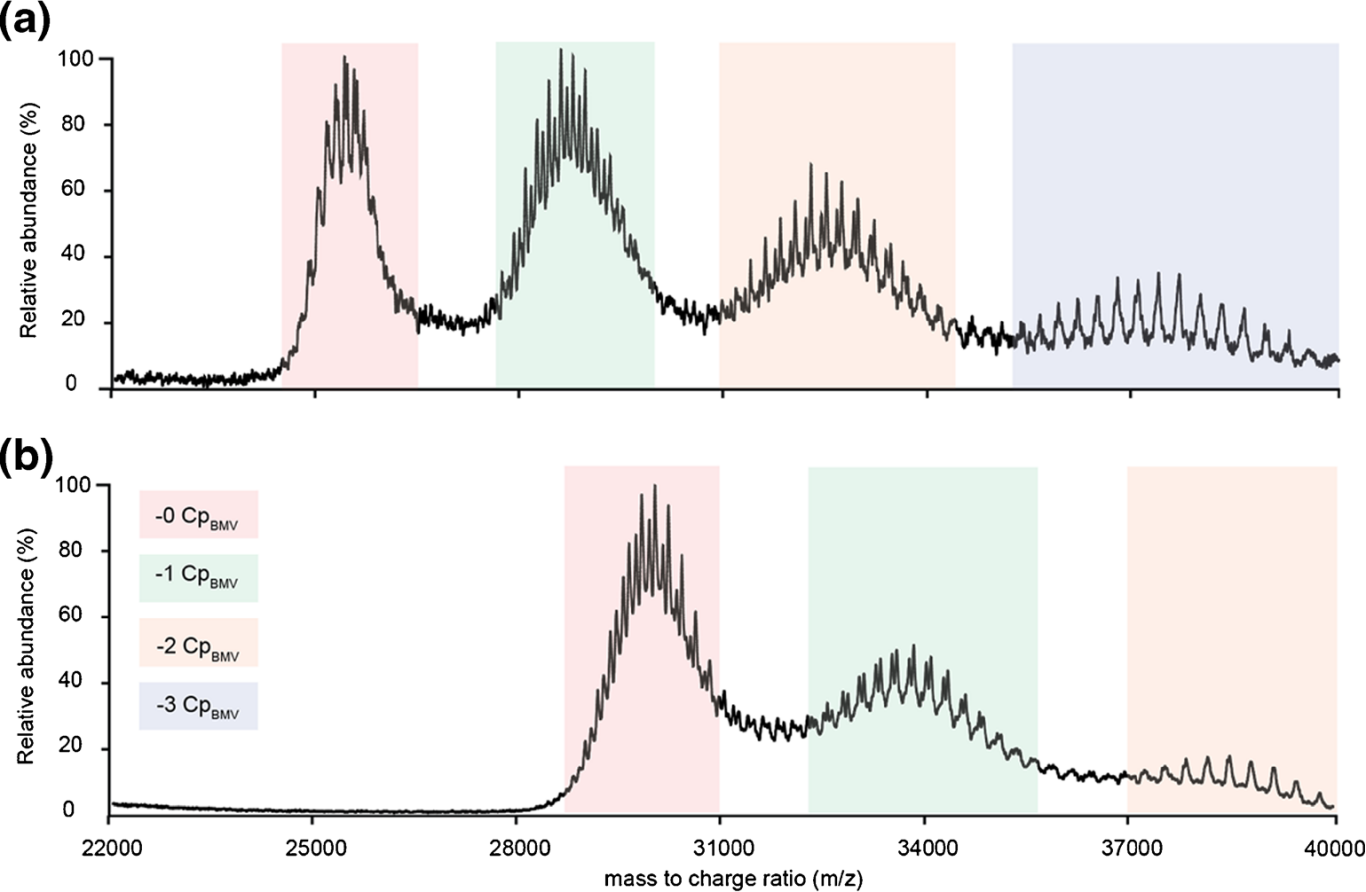
Tandem MS spectra acquired on a quadrupole time-of-flight instrument of BMV sprayed from aqueous ammonium acetate (**a**) or an ammonium acetate–triethylammonium acetate mixture (**b**). Collision induced dissociation results in sequential loss of Cp_BMV_ subunits, resulting in distinct product ions that are colored in the spectrum. The precursor signal is colored red

Up to this point, we succeeded to confidently identify RNA2 and RN3 + 4 loaded BMV virions. In an ultimate attempt to identify RNA1 particles, we performed tandem MS experiments on both BMV sprayed from aqueous ammonium acetate or an ammonium acetate/triethylammonium acetate mixture (Figure 4). Tandem MS, in combination with collision induced dissociation, is a commonly used tool in native mass spectrometry of protein complexes. It assists in identifying components of the complex by making use of the principle of asymmetric charge partitioning. As the protein complex ions collide with the inert gas molecules, single subunits are unfolded and ejected, appearing at low *m/z* (the low mass product). The high mass product, essentially the complex missing the ejected subunit, moves to higher *m/z* as it has a reduced charge density. For virus capsids, generally homo-oligomers, loss of multiple monomeric subunits is often detected, resulting in high mass products with increasingly higher *m/z*. As the quadrupole selection efficiency of ions with close to 30,000 *m/z* is rather poor, we had to perform the CID of the TEAA treated samples by selecting the full charge state envelope. Loss of up to three Cp_BMV_ subunits was detected for the ammonium acetate samples, whereas in the triethylammonium acetate samples it was limited to loss of two subunits, primarily because the maximum collision energy was reached. Although especially in the products around 33,000 *m/z* there seemed to be multiple species present, only two could be confidently assigned as RNA2 and RNA3 + 4.

**Figure 5:**
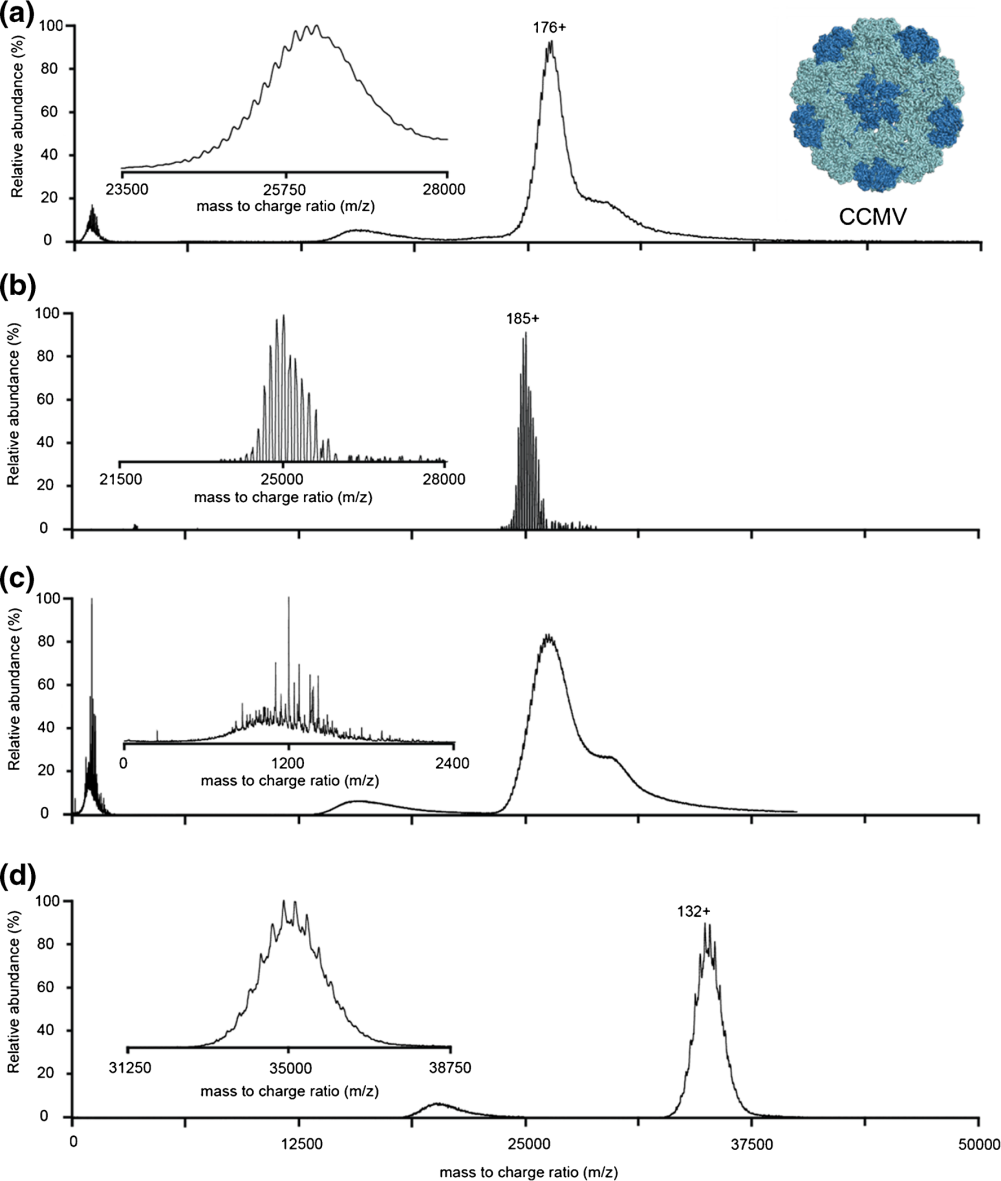
Native mass spectra of multipartite CCMV endogenous virions acquired under different experimental conditions using different mass analyzers. (**a**) Spectrum acquired using a quadrupole time-of-flight instrument. A single series of resolved charge states can be resolved at high collisional activation. (**b**) Spectrum acquired on an Orbitrap EMR with extended mass range, displaying baseline resolved ion signals. Similar to previously reported spectra, only a single series of charge states can be resolved, although with considerably higher resolution than the equivalent time-flight spectrum. (**c**) All ion fragmentation of CCMV on the quadrupole time-of-flight instrument does not show the characteristic charge reduction of asymmetric charge partitioning. Surprisingly, the preferred fragmentation pathway appears to be capsid protein backbone fragmentation. (**d**) Quadrupole time-of-flight spectrum after charge reduction using triethylammonium acetate. A more clearly resolved series of charge states is detected. Additionally, at least one additional poorly resolved series of peaks becomes visible

**Figure 6:**
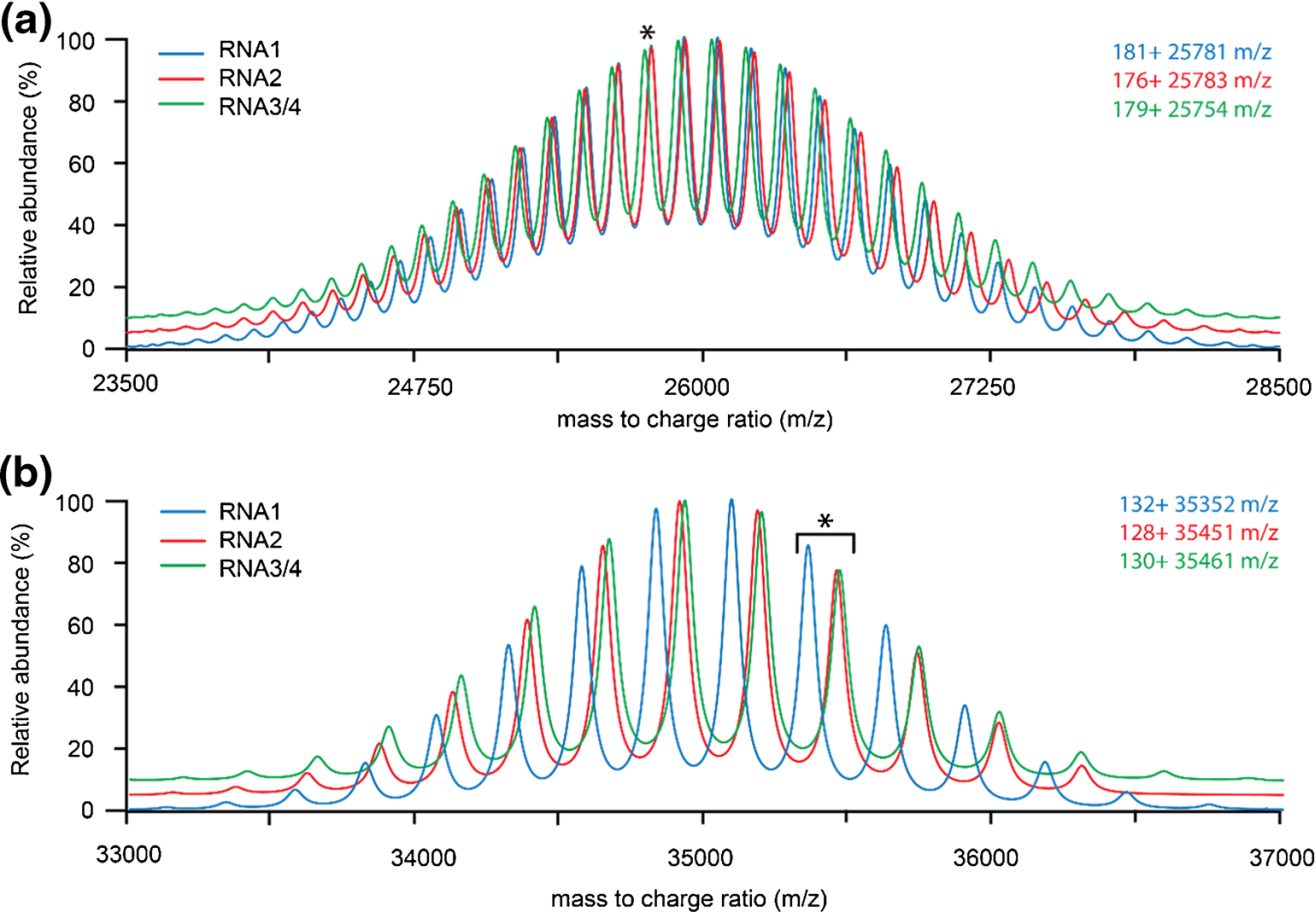
SOMMS mass spectrum simulations of CCMV using the expected masses from Table 1. Spectra are independently simulated for RNA1 (blue), RNA2 (red), and RNA3 + 4 (green) particles. The position and charge state of peaks labeled with an asterisk are added in the top right corner. (**a**) Simulation around 26,000 *m/z*, similar to data in Figure 5a and b. The mass difference between RNA1 and RNA2 (128,700 Da) is almost exactly five times the *m/z* position of the labeled peaks. Therefore, the RNA1 181+ charge state overlaps significantly with the RNA2 176+. The resolution required to resolve the different particles is higher than is feasible with the current generation of mass analyzers. (**b**) Simulation around 35,000 *m/z*, similar to data in Figure 5d. Near infinite resolution is required to resolve RNA2 and RNA3 + 4 particles. RNA1 particles are better positioned but the ability to resolve them also depends on their relative intensities

As described above, BMV and CCMV are highly similar viruses, both structurally and genomically. Therefore, we continued our native MS experiments on endogenous CCMV virions using a Q-TOF mass spectrometer. We detected a relatively broad charge state envelope around 26,000 *m/z*, which is in good agreement with estimations based on a 4.5 MDa particle (Figure 5a). In addition, we analyzed the sample under similar conditions in an Orbitrap EMR with extended mass range (Figure 5b). Both Q-TOF and Orbitrap spectra shown here of CCMV only show a single series of charge states in the spectrum, in agreement with what we previously reported [2]. Based on the expected masses in Table 1, we simulated mass spectra of the three CCMV particles theoretically present in the spectrum (Figure 6a). This clearly shows that at the *m/z* position where CCMV ion signals are located, around 26,000 *m/z*, resolving the three types of particles that are putatively present requires a higher mass resolving power than is feasible with the current generation of instruments. This requirement for high resolution is not so much a result of small mass differences between the particles as it is due to significant overlap of the charge state positions of the three particles. Therefore, we hypothesized that shifting the different species to a *m/z* other than 26,000 could allow a better separation of the individual types of particles. To achieve asymmetric charge partitioning, all-ion fragmentation of CCMV was performed using over 250 V collisional activation in the Q-TOF’s Xenon filled collision cell (Figure 5c). Surprisingly, inspection of the low *m/z* region of the spectrum (inset) indicated that instead of unfolding and ejection of Cp_CCMV_ monomers, backbone fragmentation was favored. Thus, very limited charge reduction was detected. Alternatively, addition of TEAA to the spray buffer resulted in an approximate loss of 25% of the charge on the CCMV particles, giving rise to signal around 35,000 *m/z*, instead of 26,000 *m/z* (Figure 5d). Although at least one clear series of charge states could be detected in this spectrum, the corresponding mass (4622 kDa) could not be assigned unambiguously to one of the expected CCMV particles. Additionally, simulated spectra at this *m/z* position (Figure 6b) showed strong overlap for the peak positions of RNA2 and RNA3/4 particles. Although peaks for RNA1 particles seem better positioned, it still depends on their relative abundances and the mass resolving power whether the different components can be distinguished.

In this paper, we explore the capabilities of separating and analyzing very large heterogeneous particles ( i.e., the endogenous multipartite virions of CCMV and BMV) using four different native mass spectrometry approaches. To our knowledge, the collection of native mass spectra of genome-containing virion samples in literature is quite limited [37]. Viral genomes are very diversely organized through nature and it is commonly accepted that each different organization provides some benefit during the lifecycle of the associated virus. Advantages of the genome segmentation as is present in multipartite viruses such as BMV and CCMV include increased stability, enhanced replication rate, and increased genome recombination. A disadvantage is the requirement of all particles for successful infection and any demands that this has on the timing of the production of the different particles during the infection cycle [38–40]. An intrinsic part of understanding multipartite virus biology is the measurement of the ratios of the different particles (genome segments) that are naturally produced during infection. Although this is commonly done through agarose gel electrophoresis, the native MS approach described here can potentially complement this data. For BMV and CCMV, these experiments can additionally confirm the co-packaging of the RNA3 and RNA4 segments. Native mass spectrometric analysis of such virions is challenging for a number of reasons. Although CCMV and BMV are highly similar, the results obtained for the two viruses showed quite some differences. One striking aspect was the gas-phase fragmentation behavior: Cp_CCMV_ backbone fragmentation in CCMV but ejection of Cp_BMV_ monomers for BMV. This can be an indication of structural differences between the protein capsids or their interaction with the genome. Regardless, it imposes an extra difficulty in the native MS analysis of CCMV as it introduces additional heterogeneity in the precursor spectrum and prevents efficient asymmetric charge partitioning in the tandem MS spectrum. The heterogeneity in the BMV and CCMV spectra is a direct result of both incomplete desolvation and declustering, and the composition of the different particles. The most efficient way to identify all the components in these samples is to increase the mass resolving power. For both BMV and CCMV, we were able to acquire base-line resolved Orbitrap mass spectra. Although this greatly increased the resolution in the BMV spectra (from ~290 to ~510 full width at half maximum), it unfortunately lowered the number of distinct particles that were resolved. Based on the theoretical masses, we estimated that to adequately resolve the different BMV particles, around 650–1000 resolution on the EMR is required. This moderate increase in resolution could potentially be achieved by improving the life-time of ions inside the Orbitrap mass analyzer, as this allows acquisition of spectra at longer transient times. Although the Q-TOF spectra were acquired with lower resolutions, we were able to distinguish three different BMV particles. Improvement of the desolvation and declustering on these instruments has the potential to increase the mass resolving power in these spectra and additionally improve the charge state assignment. The Orbitrap spectra of BMV appeared at lower *m/z* than their associated Q-TOF spectra, where the peak overlap was nearly complete, indicating that an increase in resolution is not the only requirement to analyze these heterogeneous megadalton assemblies. Especially interesting is the comparison with spectra acquired previously on adeno-associated virus 1 (AAV1) [2]. With masses of approximately 3.7 MDa but heterogeneities of only 7 kDa (0.19%), this sample would be highly challenging, but analysis is moderately simplified as the *m/z* shift caused by the heterogeneity is lower than the *m/z* differences between two charge states, yet resolvable by the mass spectrometer. A way to avoid this overlap of charge states is to make the analysis independent of the charge state assignment. This can be done through a number of alternative techniques, including charge detection mass spectrometry [18, 41–43], gas-phase electrophoretic mobility molecular analysis (GEMMA) [44–46], and nano(electro)mechanical-mass spectrometry (NEMS) [47–50]. Although these techniques allow analysis of a range of analyte masses and sizes, the mass heterogeneities in the BMV and CCMV sample are currently too small to be detected, emphasizing the need to expand mass heterogeneity limits [46]. For native mass spectrometry, charge reduction, either through addition of proton-scavengers or through asymmetric charge partitioning in CID, can extend these limits. Addition of the low amounts of triethylammonium acetate did not impose any additional mass spectrometric difficulties and did not require adaptation of the commonly used sample preparation methods. Acquisition of tandem MS spectra remained possible for the megadalton BMV particles, and detection of the charge-reduced products (up to 40,000 *m/z*) was not limited by the detection of the time-of-flight mass analyzer but merely the maximum collision energy available. Although no new components could be identified, the RNA2 and RNA3 + 4 particles were well resolved and their detection and relative abundance in the MS1 spectrum could be confirmed.

In conclusion, the analysis of BMV and CCMV as reported here is indicative of the current challenges in mass spectrometry of heterogeneous high molecular weight complexes, but also shows the advances and potential of the native mass spectrometry toolkit. The mass resolving power achieved on these high molecular weight assemblies is already very close to that required to successfully resolve and quantify the components in a heterogeneous mixture of virions.

## Electronic supplementary material

Below is the link to the electronic supplementary material.

Supplementary Figure S1 (DOCX 412kb)

Supplementary Figure S2 (DOCX 449kb)

Supplementary Figure S3 (DOCX 251kb)

Supplementary Figure S4 (DOCX 199kb)
