# Supplementary material for: Examining the Heterogeneous Genome Content of Multipartite Viruses BMV and CCMV by Native Mass Spectrometry
Source: J Am Soc Mass Spectrom. 2016 Feb 29;27:1000–9. doi: 10.1007/s13361-016-1348-6 (PMC4869746; doi:10.1007/s13361-016-1348-6)
Supplement: Supplementary file 2 — (DOCX 449kb) [file 13361_2016_1348_MOESM2_ESM.docx]

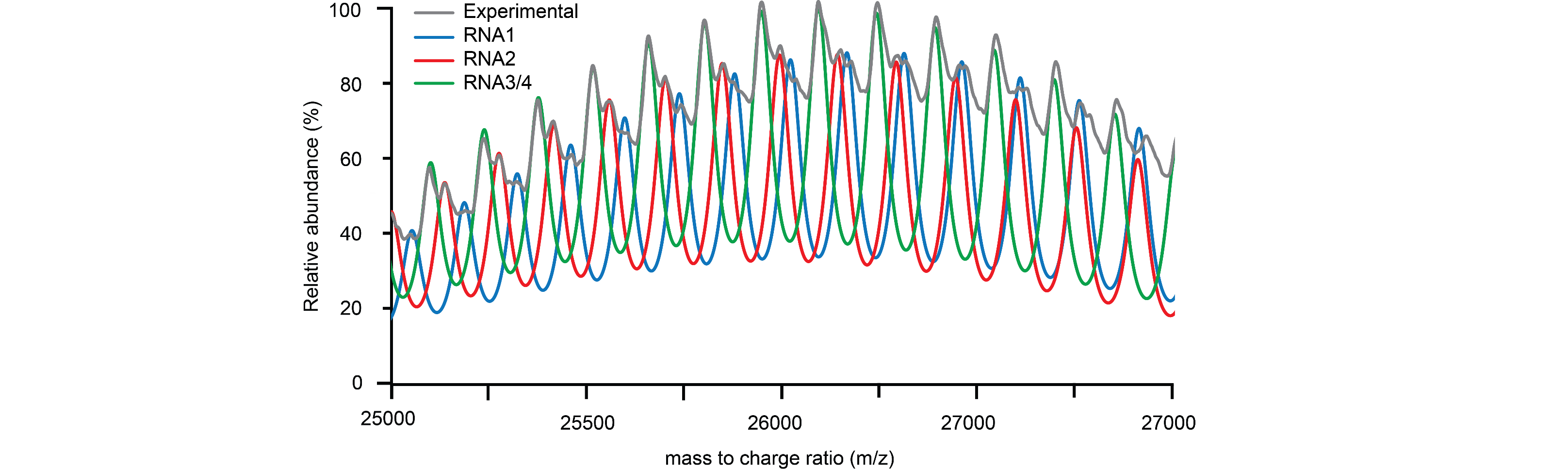


**Supplemental figure S2.** Simulation and comparison of the masses measured for BMV in ammonium acetate on the quadrupole time-of-flight instrument. The spectra are simulated for the three particles separately and combined.
