# Supplementary figures and images for: Examining the Heterogeneous Genome Content of Multipartite Viruses BMV and CCMV by Native Mass Spectrometry
Source: J Am Soc Mass Spectrom. 2016 Feb 29;27:1000–9. doi: 10.1007/s13361-016-1348-6 (PMC4869746; doi:10.1007/s13361-016-1348-6)

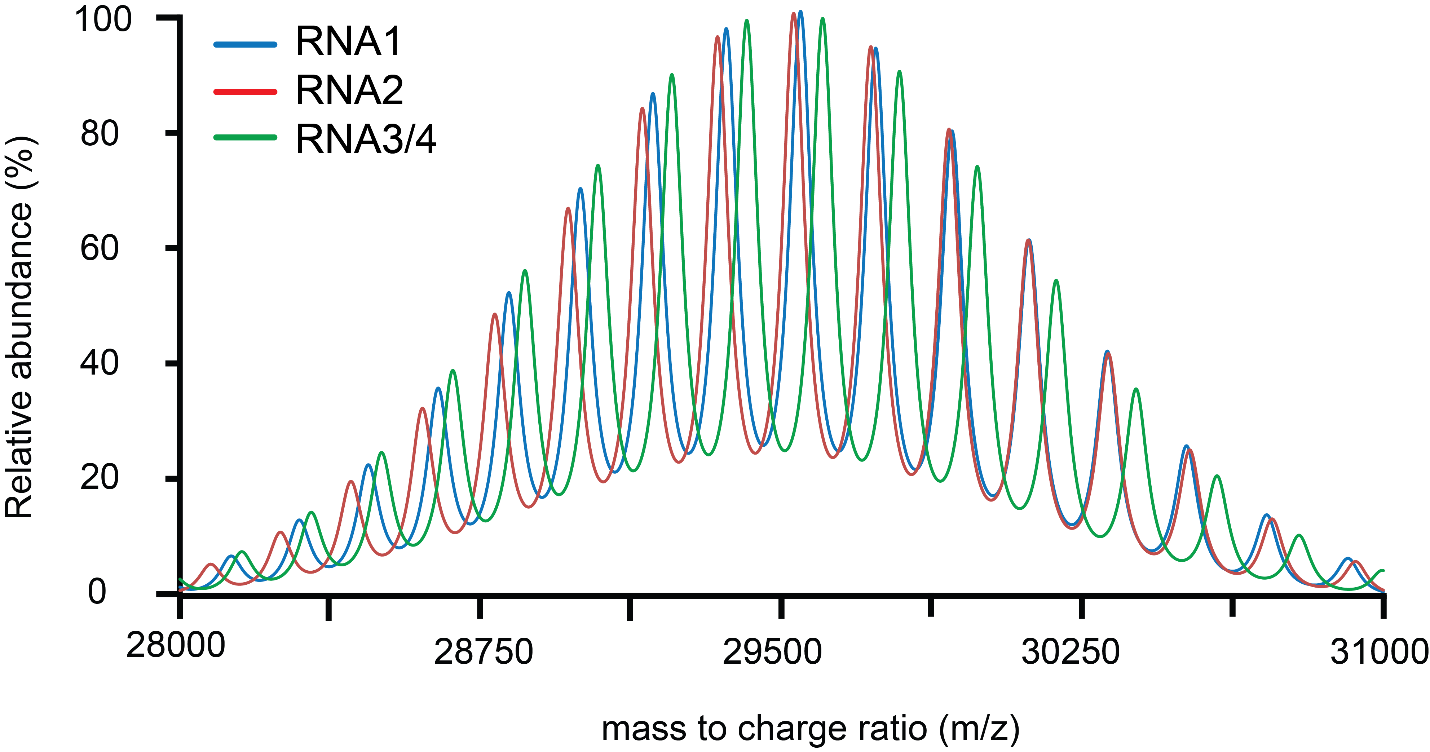


**Supplemental figure S3.** Simulation of the theoretical masses of BMV at high mass to charge ratio.

Supplement: Supplementary file 3 — (DOCX 251kb) [file 13361_2016_1348_MOESM3_ESM.docx]
