# Supplementary material for: Examining the Heterogeneous Genome Content of Multipartite Viruses BMV and CCMV by Native Mass Spectrometry
Source: J Am Soc Mass Spectrom. 2016 Feb 29;27:1000–9. doi: 10.1007/s13361-016-1348-6 (PMC4869746; doi:10.1007/s13361-016-1348-6)
Supplement: Supplementary file 4 — (DOCX 199kb) [file 13361_2016_1348_MOESM4_ESM.docx]

**Supplementary figure S4.** Agarose gel of endogenous BMV genomes shows distinct bands for the different RNA segments. Lane 1: RNA marker for size comparison. Lane 2: 500 ng of virus. Lane 3: 1500 ng of virus.

**
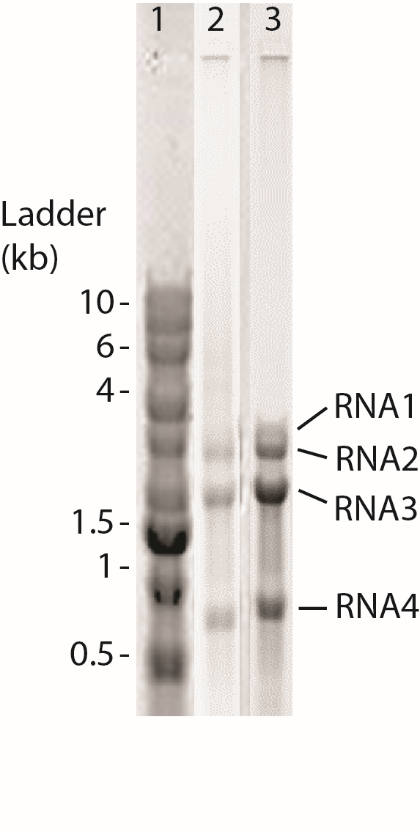
**
